# Highly Efficient Mesoporous Core-Shell Structured Ag@SiO_2_ Nanosphere as an Environmentally Friendly Catalyst for Hydrogenation of Nitrobenzene

**DOI:** 10.3390/nano10050883

**Published:** 2020-05-03

**Authors:** Bonan Zhao, Zhipeng Dong, Qiyan Wang, Yisong Xu, Nanxia Zhang, Weixing Liu, Fangning Lou, Yue Wang

**Affiliations:** Key Laboratory of Biomedical Functional Materials, School of Sciences, China Pharmaceutical University, Nanjing 211198, China; bernanzhao@163.com (B.Z.); dzp_hj@126.com (Z.D.); 18168066085wqy@gmail.com (Q.W.); 0597139@163.com (Y.X.); znx124@163.com (N.Z.); lwxcpu@163.com (W.L.); ninglfn@163.com (F.L.)

**Keywords:** mesoporous, Ag@SiO_2_, catalyst, hydrogenation, nitrobenzene

## Abstract

The size-uniformed mesoporous Ag@SiO_2_ nanospheres’ catalysts were prepared in one-pot step via reducing AgNO_3_ by different types of aldehyde, which could control the size of Ag@SiO_2_ NPs and exhibit excellent catalytic activity for the hydrogenation of nitrobenzene. The results showed that the Ag core size, monitored by different aldehydes with different reducing abilities, together with the ideal monodisperse core-shell mesoporous structure, was quite important to affect its superior catalytic performances. Moreover, the stability of Ag fixed in the core during reaction for 6 h under 2.0 MPa, 140 °C made this type of Ag@SiO_2_ catalyst separable and environmentally friendly compared with those conventional homogeneous catalysts and metal NPs catalysts. The best catalyst with smaller Ag cores was prepared by strong reducing agents such as CH_2_O. The conversion of nitrobenzene can reach 99.9%, the selectivity was 100% and the catalyst maintained its activity after several cycles, and thus, it is a useful novel candidate for the production of aniline.

## 1. Introduction

Aniline (AN), one of the important intermediates for fine chemicals such as agrochemicals, pharmaceuticals and dyestuffs, is mainly produced by selective hydrogenation of the corresponding nitrobenzene (NB) using the chemical reagent reduction method and catalytic hydrogenation method [1,2]. The chemical reduction method includes an iron powder reduction method and a sulfur reduction method, which have the advantages of being selective and are easy to operate on a small scale in a lab. However, there are serious environmental problems in these methods mentioned above. The former produces a large amount of iron waste and wastewater, while the latter causes serious pollution due to the sulfur itself. In addition, the two methods can not completely convert the substrate and the product needs to lastly be purified. With more concern about environmental issues in the chemical industry process, these two methods are being phased out by the industry [3,4,5].

To improve the catalytic hydrogenation reactivity of aromatic nitro compounds, the chemical or physical modification of the catalyst has been focused on achieving the most satisfying results. At present, catalysts applied in the field of hydrogenation of aromatic nitro compounds include metal Co, Ni, Pd, Pt, Ru, Ir, Ag [6,7]. Co and Ni catalysts have advantages in terms of cost, but the catalytic activity is relatively poor. Pd catalyst has good catalytic activity, but Sn^4+^ is required for modification, and Pt, Ru, Ir catalysts are composed of the corresponding metal nanoparticles. With a suitable oxide carrier, they usually have superior activity. However, these four noble metals are limited from applications due to the high cost.

Nanoscience has made Ag into an effective catalyst which is especially used in the hydrogenation of nitro-substituted aromatic compounds at a relatively low cost [3]. However, normally, the highly dispersible Ag nanoparticles tend to aggregate during the catalytic process and are difficult to be separated from the reaction system, which can cause lower catalytic performance and waste. Some publications reported that it had a promising catalytic activity after coating silica, which may open a new route for the preparation of aniline with nitrobenzene [8,9]. The methods for preparing Ag@SiO_2_ are mainly reported as being the following [10,11,12]. The seed growth method is to fix the synthesized metal nanoparticles on the surface of the substrate as a seed. Since Ag colloid is easy to prepare to be used as the seed in the synthesis, it makes this method have a fatal disadvantage that the Ag nanoparticles eventually exist in the product as impurities, which will confuse the properties of Ag@SiO_2_ core-shell structure. Another ultrasonic chemistry method mainly uses the cavitation of ultrasonic waves. This method needs to be carried out under less oxygen or anaerobic conditions, which requires harsh reaction conditions. The third electroless plating method is that a metal ion is reduced by a reducing agent in a solution and deposited on a surface of a catalytically active object to form a metal or alloy plating layer. However, ordinary electroless plating is complicated and time-consuming and generally uses a toxic reducing agent. This is a fast process in the inhomogeneous coverage of the Ag@SiO_2_ surface, so a large amount of Ag colloids will aggregate in the solution.

For all the defects of Ag@SiO_2_ mentioned above, its preparation method and catalytic ability need to be much improved. Especially as an effective catalyst, we should be more concerned about its behavior to transport or diffuse the reactants. Wang et al. and Chi et al. have synthesized the micron-Ag@SiO_2_ catalyst in 4-nitrophenol reduction. Big core size (over 200 nm) and great shell thickness (over 50 nm) will affect the catalytic activity compared to the data in Table 1, despite the article claiming that it showed a comparable catalytic activity with spherical silver nanoparticles [13,14]. Herein, we discovered a simple one-pot method to get a size controllable mesoporous Ag@SiO_2_ NPs, where the silver core was a substantially fixed interior. Cetyltrimethyl ammonium bromide (CTAB)-capped silver nanoparticles were used as a template to form silver-silica nanoparticles, with tetraethyl orthosilicate (TEOS) as the silica source. Distinct from the typical Stöber method with several steps [15], the entrapped nanoparticles directly reduced by aldehydes [3] can be protected from aggregation and interact with the infiltrated nitrobenzene NB, which was potentially more effective to improve the reduction of NB. Moreover, it could be recycled easily in assembly as a potential environment-friendly catalyst.

## 2. Materials and Methods

### 2.1. Materials

NH_3_·H_2_O (25%), tetraethyl orthosilicate (TEOS), CTAB were of analytical reagent grade and purchased from Shanghai Chemical Reagent Co., Ltd (Shanghai, China); silver nitrate (AgNO_3_), formaldehyde(CH_2_O), acetaldehyde(C_2_H_4_O), propionaldehyde(C_3_H_6_O), benzaldehyde (C_7_H_6_O), cyclohexane(C_6_H_6_), and hydrazine hydrate(N_2_H_4_·H_2_O) were of analytical reagent grade and purchased from Sinopharm Chemical Reagent Co., Ltd., (Shanghai, China). Igepal CO-520 (Poly(oxyethylene) (5) nonylphenyl ether) was purchased from Shanghai Aladdin Biochemical Technology Co., Ltd. (Shanghai, China). All of these chemicals were used without further purification. All solutions were produced with ultrapure water, produced by Smart-S2 DS (Nanjing Yipu-Yida Technology Development Co., Ltd., Nanjing, China) in our laboratory.

### 2.2. Fabrication of the Ag@SiO_2_ Mesoporous Nanoparticles

Silver nanoparticles were prepared by the reduction of AgNO_3_ by the high or low concentration of CH_2_O, C_2_H_4_O, C_3_H_6_O or C_7_H_6_O with the protection of CTAB. Generally, 0.4 g CTAB and 3 mL NH_3_·H_2_O (25 wt%) were dissolved in 150 mL deionized water and were heated to 60 °C for 30 min. Then, 3 mL (1.0 M) AgNO_3_ and 1 mL (1.0 M/0.1 M) CH_2_O, C_2_H_4_O, C_3_H_6_O or C_7_H_6_O were added to the solution dropwise under magnetic stirring and allowed to react for 30 min at 60 °C. Next, 2 mL silica precursor, TEOS, was added dropwise under mechanical stirring at 40 °C. After reacting for 8 **h**, it was washed with anhydrous ethanol (100 mL) and deionized water (100 mL), and the obtained particles were dried at 50 °C in vacuo.

### 2.3. Fabrication of the Ag@SiO_2_ Without-Mesoporous Nanoparticles

25 g Igepal CO-520 and 3 mL (1.0 M) AgNO_3_ were added to 200 mL C_6_H_12_. Some flocs appeared after centrifugation. 100 μL NH_2_NH_2_ was added dropwise under mechanical stirring after removing the flocs. After reacting for 0.5 h, 3 mL NH_3_·H_2_O (25 wt%) and 2 mL TEOS were added under stirring vigorously for 5 min. After reacting for 8 h, it was washed with anhydrous ethanol (100 mL) and deionized water (100 mL), and the obtained particles were vacuum dried at 50 °C.

### 2.4. Characterization of Ag@SiO_2_

Morphology of the synthesized nanoparticles were characterized by high-resolution analytical transmission electron microscopy (TEM, JEM-2100, JEOL, Tokyo, Japan) and scanning electron microscope (SEM, S-3400N II, Hitachi, Tokyo, Japan). The specific surface areas (SSA) were measured with nitrogen adsorption-desorption isotherm via the Brunauer–Emmett–Teller (BET) method. Prior to the measurements, the samples were degassed at 180 °C for 3 h. The average pore sizes, total pore volumes and pore size distributions were obtained with nitrogen desorption data, using the Barrett-Joyner-Halenda (BJH) model. Both were carried out with a Micromeritics Tristar II 3020 (Micromeritics Instrument Corp., Norcross, GA, USA), using nitrogen gas as an adsorbate at 77 K. The phase composition and crystal structure of the synthesized particles were determined by X-ray diffraction (XRD, PANalytical X’Pert Powder Pro, Malvern Panalytical, Almelo, The Netherlands) using Cu K_α1/2_ radiation (λ_α1_ = 1.5406 Å) in the range of 10°–90°(2θ). A standard silicon was measured to determine the instrumental broadening, in order to calculate the average crystallite sizes of silver based on the XRD data using Scherrer’s equation [16]. Analysis of the XRD patterns was performed using X’Pert HighScore Plus Software (Malvern Panalytical, Almelo, The Netherlands).

### 2.5. Catalytic Test

The hydrogenation of nitrobenzene (NB) was employed as a probe reaction to test the activity of the Ag@SiO_2_ catalyst that was prepared from different types and different concentrations of aldehydes. In each trial, 0.5 g nitrobenzene was used as substrate, hydrogen was used as reducing agent, 50 mg Ag@SiO_2_ nanoparticle was used as catalyst and stainless steel was chosen to be the reaction vessel in the catalytic studies. The reaction pressure was 2.0 MPa and the temperature was 140 °C. After reacting for 6 h, the conversion was detected by a gas chromatography-mass spectrometer (GCMS-2010, Shimadzu, Kyoto, Japan). The recycling test was also carried out 10 times.

## 3. Results and Discussion

### 3.1. Preparation and Morphology of Catalysts

**Figure 1:**
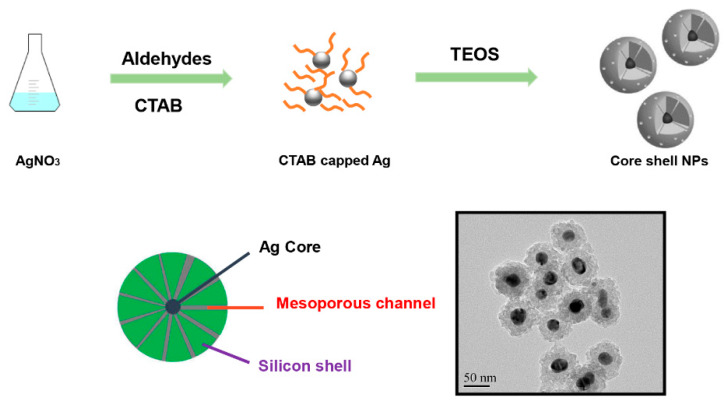
Graphical scheme of the fabrication of Ag@SiO_2_ nanoparticles with a single silver core.

**Figure 2:**
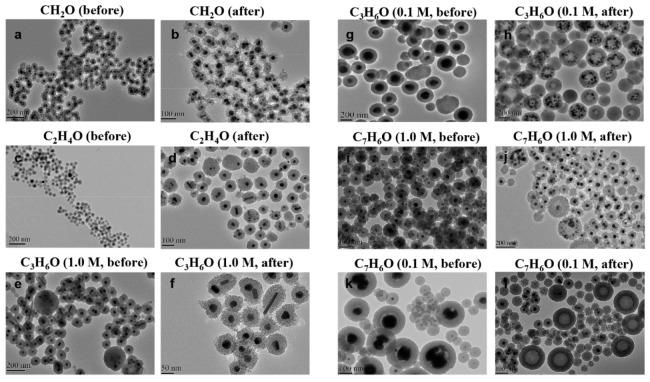
TEM images of Ag@SiO_2_ nanoparticles prepared by CH_2_O before (**a**) and after (**b**), C_2_H_4_O before (**c**) and after (**d**), C_3_H_6_O (1.0 M) before (**e**) and after (**f**), C_3_H_6_O (0.1 M) before (**g**) and after (**h**), C_7_H_6_O (1.0 M) before (**i**) and after (**j**), and C_7_H_6_O (0.1 M) before (**k**) and after (**l**) nitrobenzene hydrogenation.

The general synthetic procedure to get silica-protected silver nanoparticles by the reduction of silver nitrate with different aldehydes [17,18,19] is shown in Figure 1. The morphology of particles prepared by different types and concentrations of aldehydes collected by TEM were shown in Figure 2. As shown in the TEM, most of the particles were monodispersed with the average core radius, which ranged from 80 nm to 150 nm, coated with the mesoporous silica. It generally had good dispersity and remarkable monodisperse core-shell structures. However, due to the different reducing ability of the aldehydes [20], the sizes of the obtained Ag core were distinct from each other. Figure 2a,c showed that the Ag core, with similar size distribution, was small and dense, which was produced by the strong reducing agent of CH_2_O or C_2_H_4_O at a concentration of 0.1 M. However, big and loose sliver dots were observed in Figure 2g,k, which were reduced by the weak reducing agent of C_3_H_6_O or C_7_H_6_O at the same concentration (0.1 M). We found that the weaker reducing agent might be accompanied by the slower reduction rate, which resulted in a slow nucleation rate and slow growth. Therefore, the Ag core particle size was inclined to be larger. Especially in Figure 2k, there were two size distribution patterns mixed, most of which were the bigger particles. However, with the increasing concentration of these weak reducing agents, the nucleation of Ag was promoted rapidly, due to the accelerated corresponding reducing rate. It was obvious that the weak reducing agent of C_3_H_6_O or C_7_H_6_O at a high concentration (1.0 M) was able to produce a similar morphology of Ag@SiO_2_ (Figure 2e,i) with Figure 2a. All in all, the reducing ability and the concentration of aldehyde are the ultimate factors to monitor the size of Ag@SiO_2_ NPs, thus, will exhibit distinct catalytic activities in the following study.

**Figure 3:**
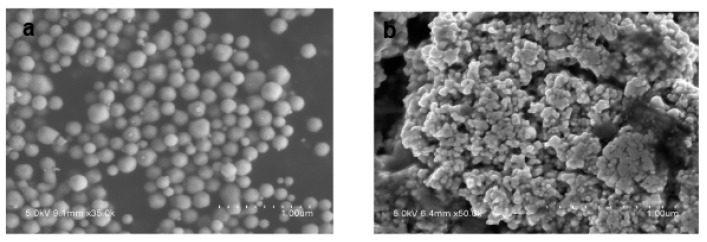
Characterization of the prepared Ag nanoparticles by SEM images: (**a**) prepared at low concentration of C_7_H_6_O, (**b**) prepared by CH_2_O.

It can be supported by the scanning electron microscopy images (Figure 3a) of the Ag core generated in a low-concentration benzaldehyde system without TEOS. The overall particle size was relatively large; even the smallest sphere in the picture was about 100 nm with small bud-shaped Ag that emerged on the surface. These bud-shaped Ag tended to grow continuously, which might have resulted in the maximum particle size being 300 nm. Figure 3b showed the generation of Ag cores in the formaldehyde system without TEOS. The overall morphology appeared to have a much smaller particle size and a spherical shape.

We speculated that during the hydrolysis of TEOS, excessive benzaldehyde will spatially hinder the condensation between Si-OH, making the rate of SiO_2_ deposition slower, while the Ag core continued to grow. This leads to a larger volume of Ag core. By contrast, perhaps due to the faster growth of Ag NP_S_ when we used a strong reducing agent such as CH_2_O, the rate of SiO_2_ deposition was faster than the former, and thus the smaller Ag cores were obtained.

### 3.2. Catalytic Performances

The catalytic activities of the as-prepared nanoparticles were assessed by the reduction of 4-nitrophenol by H_2_ with the different catalysts in aqueous solution. Table 1 presented the results for the synthesis of aniline by different kinds and concentrations of aldehydes under high pressure. The conversion of nitrobenzene (NB) was 99.9% in the case of Ag@SiO_2_ prepared by the relatively strong reducing agent of CH_2_O, C_2_H_4_O. When using the Ag@SiO_2_ prepared by the weak reducing agent of C_3_H_6_O or C_7_H_6_O, the conversion of NB reached 98.6% and 97.3% at high concentration (1.0 M), but only reached 11.5% and 10.1% at low concentration (0.1 M). Figure 2 showed the characterization of the prepared nanoparticles after the nitrobenzene hydrogenation by TEM images. Compared with the morphology before the reaction, the outer layer of SiO_2_ in Figure 2b,d were obviously loose, but the Ag core was still kept in the interior. Similar results were obtained in Figure 2f,j. Meanwhile, Figure 2h,l depicted that almost all the big Ag cores disappeared and some of them reformed the smaller scattered Ag in the shell. Compared with the former, these bigger Ag cores had weak catalytic performance but diffused more reduced by C_3_H_6_O and C_7_H_6_O at low concentration. Some of the Ag cores were even completely removed from the SiO_2_ shell after hydrogenation reactions.

**Figure 4:**
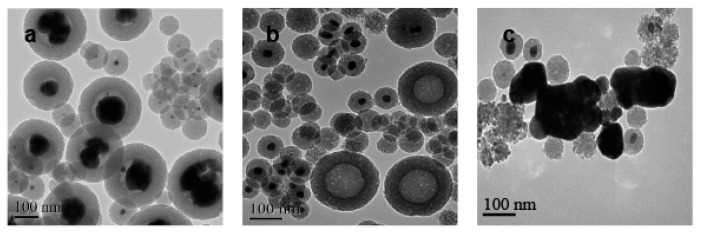
Characterization of the prepared Ag@SiO_2_ nanoparticles by TEM images: prepared at low concentration of C_7_H_6_O (**a**) before and (**b**,**c**) after nitrobenzene hydrogenation.

For the Ag@SiO_2_ obtained from a low concentration of benzaldehyde, Figure 4a showed the morphology of Ag@SiO_2_ NPs before adding H_2_. We found that it contained two kinds of particle diameters. One was the particle with a diameter of about 100 nm; the majority of the particles were about 250 nm bearing large Ag nuclei. Figure 4b showed the morphology of these Ag@SiO_2_ NPs after the nitrobenzene hydrogenation and the silver cores of large particles were almost completely diffused, while the Ag cores of the small particles still existed. The experimental results in Figure 4c showed that some Ag particles outside the shell can be found, which should be generated from the diffused bigger Ag cores and then re-aggregated after the reaction. In fact, it was true that the Ag NPs without SiO_2_ shell had a very low catalytic activity, which coincided with the low catalytic activity result of this Ag@SiO_2_ (Table 1). It revealed that the diffusion process might occur before the catalytic reaction.

We also calculated the size distribution via size deviation [15] in Table 1. The average sizes of the Ag@SiO_2_ particles based on the TEM images with standard deviations were found to be 85 ± 6 nm, 81 ± 7 nm, 102 ± 2 nm, 130 ± 37 nm, 105 ± 13nm and 142 ± 50 nm, which confirmed that the much narrower size distribution in case of CH_2_O is a further benefit of effective catalytic activity.

Moreover, the above experimental results showed an interesting phenomenon. The shells of Ag@SiO_2_ with high catalytic efficiency all became loose after the reaction, while the main outer layer of the particles of large-sized Ag core was still dense. However, nearly all the small Ag cores were still kept inside the looser shell after the reaction. However, most of the larger Ag cores diffused to the shell; some of them even went outside the dense shell (Table 1). This unexpected and contradictory phenomenon reminded us that the stability of the Ag core might not be related directly to the nitrobenzene hydrogenation itself. This phenomenon suggested that the diffusion of the Ag core should not be occurring after the completion of the catalytic reaction, otherwise the Ag core should overflow more easily in the loose shell particles after the reaction.

**Figure 5:**
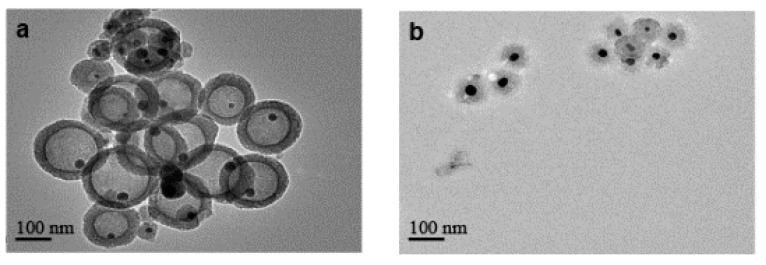
Characterization of the prepared nanoparticles without substrate after reaction under argon by TEM images: (**a**) prepared by low concentration of C_7_H_6_O, (**b**) prepared by CH_2_O.

In order to prove our assumption, no substrate was added and the hydrogen gas was replaced with argon. Then, we repeated the same process (reaction pressure was 2.0 MPa, temperature was 140 °C and reaction time was 6 h) as described in Section 2.5. From the result shown in Figure 5a, we found that the bigger Ag cores which were prepared at a low concentration of C_7_H_6_O still became smaller or disappeared completely from the interior. However, small Ag cores prepared by CH_2_O in Figure 5b was always kept inside the core-shell structure. This result indicated that the silver core overflow was independent of the nitrobenzene hydrogenation.

**Figure 6:**
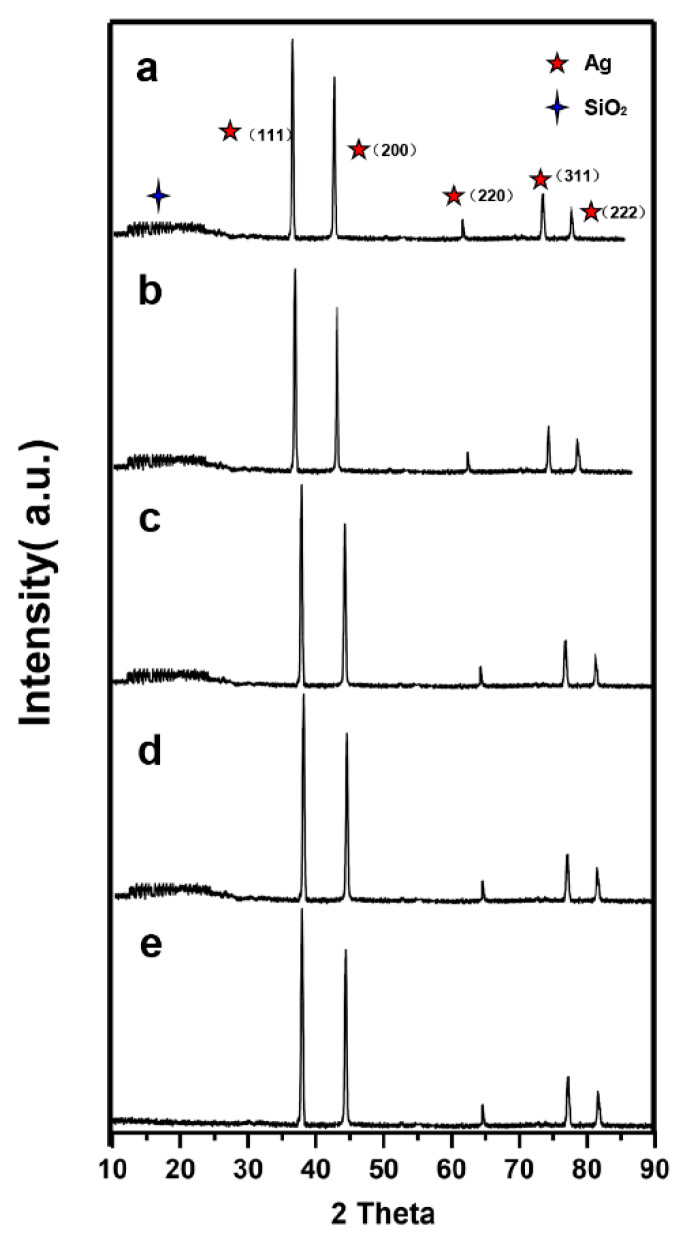
XRD patterns of a, b, c, d and e samples. (**a**) Ag@SiO_2_ prepared by CH_2_O before reaction, (**b**) Ag@SiO_2_ prepared by C_7_H_6_O at low concentration before reaction, (**c**) Ag@SiO_2_ prepared by CH_2_O after reaction, (**d**) Ag@SiO_2_ prepared by C_7_H_6_O at low concentration after reaction, (**e**) Ag NPs prepared by CH_2_O without adding tetraethyl orthosilicate (TEOS).

In Figure 6, the XRD pattern showed that five diffraction peaks assigned to metallic Ag are found in the spectrum of Ag@SiO_2_. The peaks at 38.1°, 44.3°, 64.4°, 77.5° and 81.8° were ascribed to Ag (111), (200), (220), (311) and (222), which are shown apparently in the XRD pattern in good accordance with standard JCPDS cards no. 04-0783. Compared with Ag NPs in Figure 6e, the broad humps in the diffraction spectra centered at 22° are due to amorphous silica particles. 

**Figure 7:**
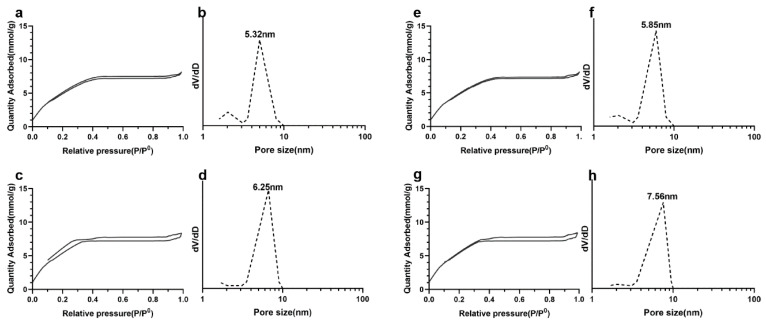
N_2_ adsorption–desorption isotherm and pore size distribution of Ag@SiO_2_. (**a**,**b**) Ag@SiO_2_ prepared by CH_2_O before reaction, (**c**,**d**) Ag@SiO_2_ prepared by C_7_H_6_O at low concentration before reaction, (**e**,**f**) Ag@SiO_2_ prepared by CH_2_O after reaction, (**g**,**h**) Ag@SiO_2_ prepared by C_7_H_6_O at low concentration after reaction.

Figure 7 presented the nitrogen adsorption–desorption isotherms and pore size distributions of the dry powders. All samples exhibit an approximate type IV isotherm pattern, as defined by IUPAC for mesopore characteristics. The pore sizes were calculated as 5.32 nm, 6.25 nm, 5.85 nm and 7.56 nm by BJH methods. Other data about measuring mean pore sizes and specific surface area by BET analysis were revealed in Table 2.

So what will influence the diffusion of the Ag core under the reaction condition? Normally, the formation of Ag^+^ which then passes through the porous shell is a reasonable explanation. In the case of this process, the substrate or H_2_ is necessary to contact Ag and induce the electron donation from the Ag core. However, actually, even without these two agents the great change of bigger Ag core occurs, which means there is no way to produce Ag^+^. So, we suppose that the other possible mechanism of Ag atom is just diffusing at this high pressure and temperature. A similar report was published by Lei et al. in 2012 [21]. For the larger surface area of the bigger Ag core, less Ag-O-Si- occupied its surface, to make Ag atom unstable and diffuse through the porous shell easily. Another phenomenon in Figure 2h, that some Ag atoms have congregated to form a new cluster in the shell, can further prove this diffusion process. Irene et al. in 2018 [22] also mentioned that once the Ag is diffusing out of the shell, its catalytic activity will be decreased greatly because of the aggregation of the catalyst. It is coinciding with the control experiment we performed using the Ag NPs as the catalyst (about 10% conversion). In contrast, the smaller Ag core with more Ag-O-Si- cross-link occupying its surface is more stable. Then, once it contacts the reagent through the porous shell, it will activate the substrate or H_2_ rapidly and produce high catalytic efficiency.

### 3.3. Catalytic Mechanism

**Figure 8:**
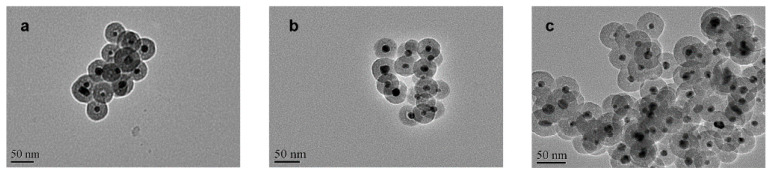
Characterization of the nanoparticles prepared by CH_2_O after reaction by TEM images: (**a**)NB/under H_2_, (**b**) without NB/under H_2,_ (**c**) NB/under Ar.

**Figure 9:**
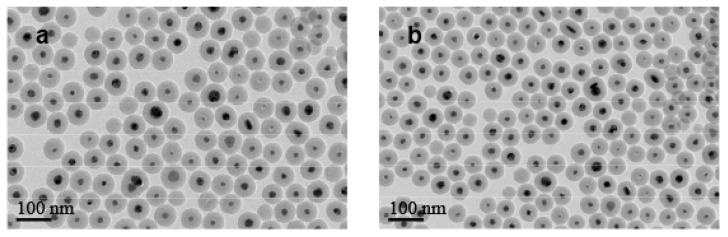
Characterization of the without-mesoporous nanoparticles prepared by reverse micelle technique by TEM images: (**a**) before and (**b**) after nitrobenzene hydrogenation.

In order to clarify the catalytic mechanism of Ag@SiO_2_ with small Ag cores_,_ we chose those NPs prepared with CH_2_O for further study. Figure 8 showed that the interior Ag nearly kept unchanged after the reaction at different reaction conditions, which revealed that the state of the silver core was independent of hydrogen or substrate. These phenomena confirmed what we concluded for the stability of Ag cores. Next, we prepared some Ag@SiO_2_ nanoparticles with dense shell structure by reverse micelle technique [23]. In Figure 9, the shape of the nanoparticles looks like a compact sphere. After 4, 6, and 12 h of catalytic reaction, the conversion was almost 0% and the morphology of the particles had no change. These results demonstrated that the adsorption of the reactants passing through the mesoporous channel was the necessary pathway to trigger the catalytic reaction.

**Figure 10:**
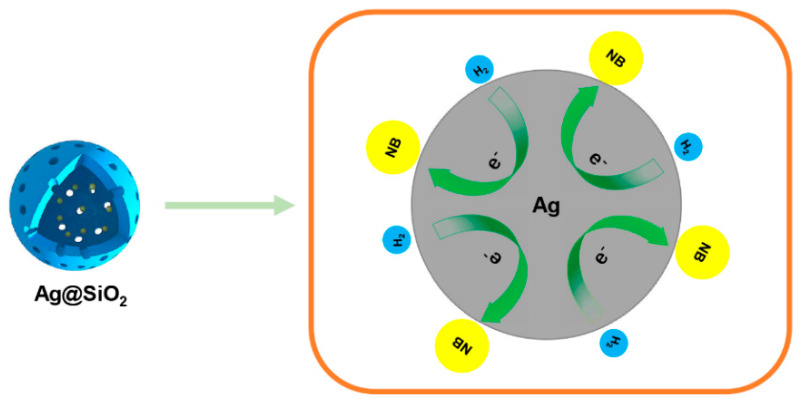
Schematic explanation of the catalytic process occurring on the nanoparticles during the reaction.

Thus, the model of the reaction can be classified as a Langmuir–Hinshelwood mechanism, and both reactants need to be absorbed on the surface prior to reaction. The mechanism of catalytic activities as illustrated in Figure 10 [24,25] as follows, where the NB is nitrobenzene, σ is the adsorption site on the Ag core surface, and AN is aniline.

NB + σ → NBσ

H_2_ + σ → H_2_σ

NBσ + H_2_σ → ANσ + H_2_Oσ

ANσ → AN + σ

H_2_Oσ → H_2_O + σ

Firstly, the reagents, nitrobenzene and hydrogen, passed through the silica shell via the pores and were absorbed on the surface of the Ag cores. Then, the electrons of the nucleophile (H_2_) transferred across the interface of Ag cores for Ag play the role of an electronic transmission medium. Then, the electrophile captured the electrons from the interface and completed conversion. Here, the rate-determining step was the reaction on the Ag core surface. The adsorption/desorption equilibrium was achieved much faster and evaluated by the Langmuir isotherm model. Silicon shell not only promoted the free diffusion of reactants towards the catalyst surface, but reduced the aggregation of the catalyst. Moreover, the attachment of SiO_2_ shell to the Ag surface could slow down the oxidation processes of Ag and improve the stability of Ag nanoparticles [21], which was more beneficial for the separation and possibly recycling of the catalyst.

**Figure 11:**
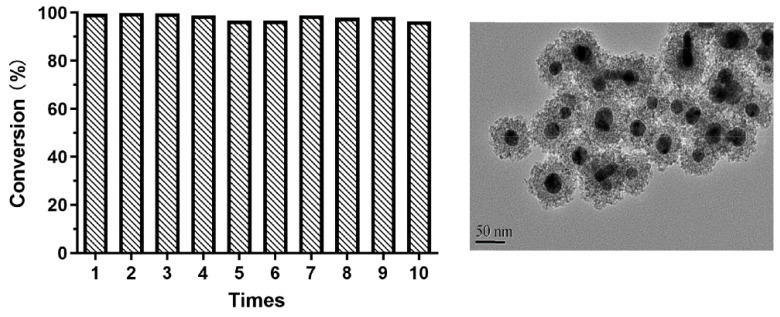
Conversion and TEM image Ag@SiO_2_ (prepared by CH_2_O) after 10 cycles of nitrobenzene hydrogenation.

The recycling test was carried out 10 times. As expected, the catalytic conversion rate remained higher than 95% in Figure 11 after the reaction was repeated ten consecutive times, because of the scarce leakage of silver due to the protection of the outer silica layer.

## 4. Conclusions

In conclusion, we have reported an Ag@SiO_2_ catalyst prepared by a one-pot method using different types of aldehydes, and some of them prepared by a strong reducing agent, such as CH_2_O, showed the best activity for hydrogenation of nitrobenzene producing the aniline. The size of the silver nanoparticles, the interaction between the silver cores and the silica support may play an important role in the catalytic reaction system. We discussed that the best catalyst may have more Ag-O-Si- cross-link occupying smaller Ag core’s surface, which was quite stable before and after the reaction. Due to the protection of the outer silica layer even after the efficient catalytic reaction, scarce leakage of silver demonstrated that this Ag@SiO_2_ is a promising material in the selective hydrogenation of nitrobenzene on a large scale. Therefore, this progress is valuable to enable the application of these Ag@SiO_2_ catalysts in energy conversion and environmental protection.

## Figures and Tables

**Table 1:**
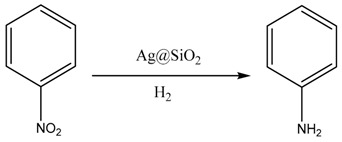
Properties of hydrogenation reactions over fresh Ag@SiO_2_ catalyst.

Entry	Conversion (%)	Selectivity (%)	Ag average Size (nm) before the Reaction	Ag average Size (nm) after the Reaction	SiO_2_ Thickness (nm) before the Reaction	SiO_2_ Thickness (nm) after the Reaction	Particle Size ± STDEV (nm)
CH_2_O	99.9	100	35.5	36.7	30.2	29.5	85 ± 6
C_2_H_4_O	99.9	100	36.2	37.5	28.7	27.9	81 ± 7
C_3_H_6_O (1.0 M)	98.6	100	51.4	52.9	37.3	32.4	102 ± 12
C_3_H_6_O (0.1 M)	11.5	100	103.6	51.2	38.7	42.7	130 ± 37
C_7_H_6_O (1.0 M)	97.3	100	53.6	54.1	36.2	31.4	105 ± 13
C_7_H_6_O (0.1 M)	10.1	100	95.7	25.0	31.4	42.3	142 ± 50

Reaction conditions: 6 h, 413 K, 2.0 MPa.

**Table 2:** Pore size and specific surface area of Ag@SiO_2_.

	Ag@ SiO_2_-a	Ag@ SiO_2_-b	Ag@ SiO_2_-c	Ag@ SiO_2_-d
Pore size (nm)	5.74	6.93	6.15	8.12
Specific surface area (m^2^ g^−1^)	375	346	342	324

(**a**) prepared by CH2O, (**b**) prepared by C7H6O at low concentration, (**c**) prepared by CH2O after nitrobenzene hydrogenation, (**d**) prepared by C7H6O at low concentration after nitrobenzene hydrogenation.
